# Overexpression and Role of HHLA2, a Novel Immune Checkpoint, in Colorectal Cancer

**DOI:** 10.3390/ijms24065876

**Published:** 2023-03-20

**Authors:** Agnieszka Kula, Miriam Dawidowicz, Sylwia Mielcarska, Paweł Kiczmer, Hanna Skiba, Małgorzata Krygier, Magdalena Chrabańska, Jerzy Piecuch, Monika Szrot, Julia Robotycka, Błażej Ochman, Bogumiła Strzałkowska, Zenon Czuba, Elżbieta Świętochowska, Dariusz Waniczek

**Affiliations:** 1 Department of Oncological Surgery, Faculty of Medical Sciences in Zabrze, Medical University of Silesia, 41-808 Katowice, Poland; 2 Department of Medical and Molecular Biology, Faculty of Medical Sciences in Zabrze, Medical University of Silesia, 19 Jordana, 41-800 Zabrze, Poland; 3 Department and Chair of Pathomorphology, Faculty of Medical Sciences in Zabrze, Medical University of Silesia, 13-15 3 Maja, 41-800 Zabrze, Poland; 4 Department of General and Bariatric Surgery and Emergency Medicine, Faculty of Medical Sciences in Zabrze, Medical University of Silesia, 41-808 Katowice, Poland; 5 Department of Microbiology and Immunology, Faculty of Medical Sciences in Zabrze, Medical University of Silesia, 40-055 Katowice, Poland

**Keywords:** colorectal cancer, immune checkpoint, HHLA2, MSI, immunotherapy

## Abstract

The study aimed to investigate correlations between HHLA2 levels and parameters, including microsatellite instability (MSI) status, CD8+ cells, and histopathological features: budding, tumor-infiltrating lymphocytes (TILs), TNM scale, grading, cytokines, chemokines, and cell signaling moleculesin colorectal cancer (CRC). Furthermore, the immune infiltration landscape and HHLA2-related pathways in colorectal cancer using available online datasets were analyzed. The study included 167 patients diagnosed with CRC. Expression of HHLA2 was detected by immunohistochemistry method (IHC) and enzyme-linked immunosorbent assay (ELISA). The IHC was used to evaluate the MSI and CD8+ status. The budding and TILs were measured using a light microscope. The concentrations of cytokines, chemokines, and cell signaling molecules were measured to analyze the data by the Bio-Plex Pro Human cytokine screening panel, 48 cytokine assay, and principal component analysis (PCA). Geneset enrichment analysis (GSEA) was conducted to identify HHLA2-related pathways. The biological function of HHLA2 was predicted by Gene Ontology (GO). Analysis of the immune infiltration landscape of HHLA2 in colorectal cancer was made by the web-based tool Camoip. High HHLA2 expression was detected in CRC tumor tissues compared to the adjacent noncancerous tissues. The percentage of HHLA2-positive tumors was 97%. GSEA and GO showed that HHLA2 upregulation correlated with cancer-related pathways and several biological functions. Tumor-infiltrating lymphocytes score correlated positively with IHC HHLA2 expression level percentage. There was a negative correlation between HHLA2, anti-tumor cytokines and pro-tumor growth factors. This study provides a valuable insight into the role of HHLA2 in CRC. We reveal the role of HHLA2 expression as well as a stimulatory and inhibitory immune checkpoint in colorectal cancer. Further research may verify the therapeutic values of the HHLA2-KIR3DL3/TMIGD2 pathway in colorectal cancer.

## 1. Introduction

Microsatellite instability (MSI) is a result of mutations in DNA mismatch repair genes including *MLH1*, *MSH2*, *MSH6*, and *PMS2*. *MSI* is found in 10% to 15% of sporadic colorectal cancers [1,2]. Microsatellite-instability-high (MSI-H) colorectal cancers are related to a high tumor mutation burden (TMB). That results in high expression of checkpoints such as programmed cell death protein 1 (PD-1), programmed death-ligand 1 (PD-L1), and cytotoxic T-lymphocyte-associated protein 4 (CTLA-4) [3,4,5].

As a consequence of microsatellite-stable (MSS) colorectal cancer’s lower TMB and a poorer level of its immune infiltration, it has previously been considered to be resistant to immunotherapy. Recently, a novel insight into the purpose of immunotherapy in MSS CRC has been presented [5,6]. The MSS colorectal cancer’s responsiveness to checkpoint blockade might be improved by combining immune modulators [5]. Nevertheless, many patients are not adequately treated at the present time. There is still a large group of patients who present resistance to immune checkpoint inhibitors therapy [7,8]. That is the reason why it is still crucial to conduct research into new inhibitory receptors for immunotherapy. Human endogenous retrovirus-H long terminal repeat-associating protein 2 (HHLA2, B7H7, B7y) is a member of the B7 family. It is thought to be a potential therapeutic approach for various cancer immunotherapies. HHLA2 has a restricted expression in normal human tissues but a broad one in human cancers [9,10].

It has been reported that HHLA2 contributes to complex functions in the tumor microenvironment [9,11,12,13]. However, the value of HHLA2 in colorectal cancer remains unknown and needs further investigation. In addition, the reports on HHLA2 are often contradictory. Zhang et al. describe low expression of HHLA2 in the majority of the 214 CRC patients in multiracial tumor microarrays [14]. In contrast to these results, Zhu et al. found an overexpression of this protein in 63 CRC and its association with worse survival. There is still much to explain in the context of this immune checkpoint in CRC. To evaluate the role of HHLA2 in colorectal cancer, we assessed its levels in relation to various parameters, including MSI status, CD8+ cells, and the histopathological features such as budding, tumor-infiltrating lymphocytes (TILs), the response of the host immune system, TNM scale, grading, cytokines, chemokines, and cell signaling molecule panels. Additionally, we analyzed HHLA2-related pathways in colorectal cancer using available online datasets.

The exact function of HHLA2 in colorectal cancer is still not fully understood.

## 2. Results

### 2.1. Overexpression of HHLA2 in CRC Tissues

**Figure 1:**
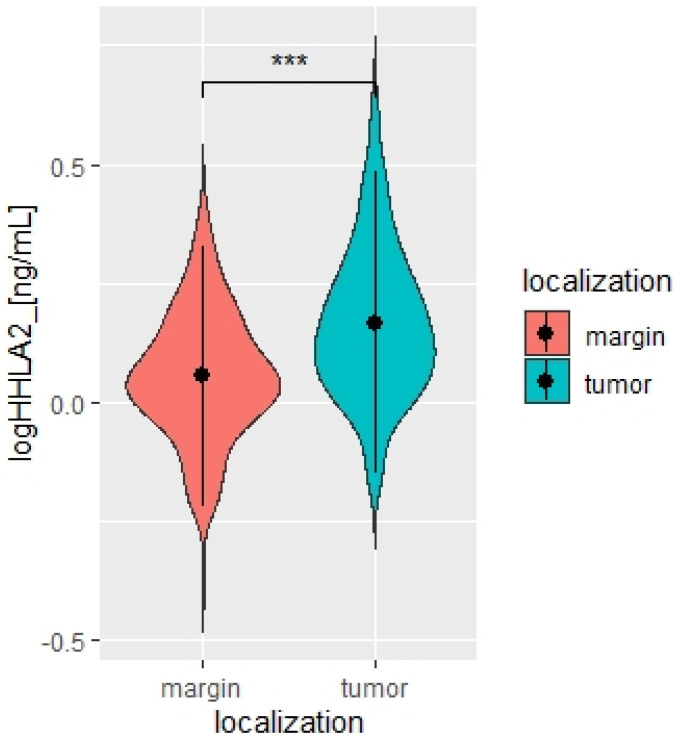
Violin plot of HHLA2 concentrations in tumor and margin tissue [ng/mL], N = 167. Paired T-test. *** *p* < 0.0001.

**Figure 2:**
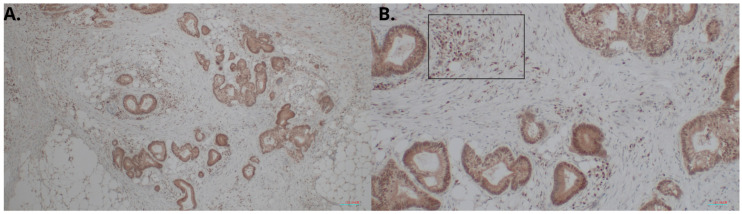
HHLA2 immunostaining in CRC specimens. Intense staining of the glands and accompanying infiltration of lymphocytes: (**A**) 100× magnification; (**B**) 400× magnification. In (**B**), HHLA2-positive lymphocytes are marked in the frame.

We measured the HHLA2 protein levels in 159 CRC and noncancerous tissues in this study by ELISA test. HHLA2 was upgraded in tumor tissues homogenates compared to the margin (*p* < 0.0001 Figure 1, Table 1). Further, expression of HHLA2 was detected by the immunohistochemistry method in 77 randomly selected tumor slides. High HHLA2 expression was found in CRC tumor tissues. The percentage of HHLA2-positive tumors was 97%. Figure 2 presents HHLA2 immunostaining in CRC specimens, with staining of the glands and accompanying infiltration of lymphocytes. Table 2 presents the division of the examined tumors into HHLA2-positive and HHLA2-negative tumors.

### 2.2. The Expression of HHLA2 in Relation to MSI/MSS Status and Clinicopathological Parameters

**Figure 3:**
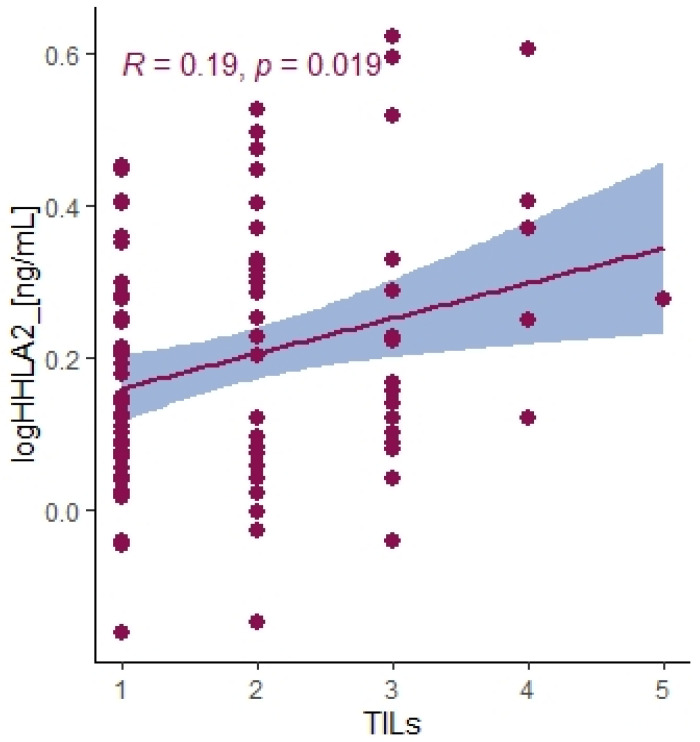
Correlation between HHLA2 concentrations in tumor tissue homogenates and TILs score. Tau Kendall correlation coefficient.

There was no relation between the HHLA2 expression and microsatellite status (Table 3). Moreover, the TILs (TILs include T cells, B cells, natural killer (NK) or dendritic cells (DC) infiltrating the tumor regions). the main response of host immune system, correlated positively with the percentage of IHC HHLA2 expression level (Figure 3). There was no association between HHLA2 and CD8+ T-cell infiltration and other clinicopathological features. We reported no association between HHLA2 expression and MSI/MSS status. We observed significant overexpression of HHLA2 both in MSI and MSS tumors (Table 3).

### 2.3. Principal Component Analysis

Our study chose the Bio-Plex Pro Human Cytokine screening panel with a biologically relevant collection of adaptive immunity molecules, chemokines, pro-inflammatory cytokines, and anti-inflammatory cytokines related to cancerogenesis. 

**Figure 4:**
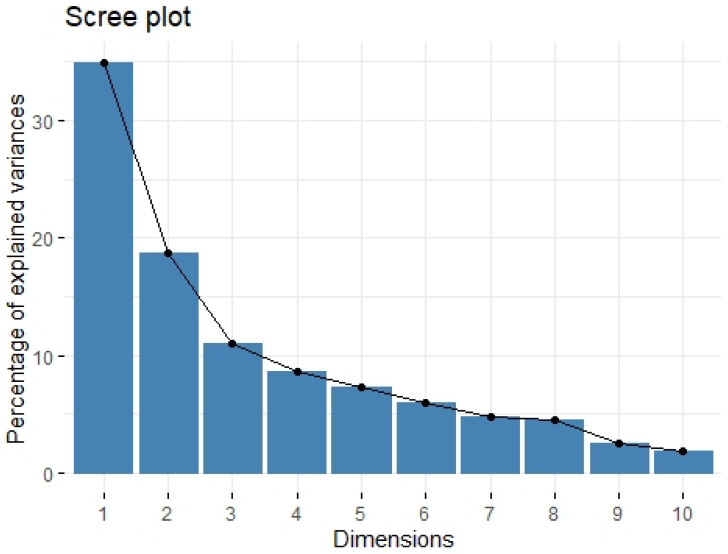
Scree plot of principal component analysis containing protumor growth factors (HGF, M-CSF, b-NGF, SCGF-b, IL-7, FGFbasic, G-CSF, PDGF-bb, VEGF, GM-CSF). Scree plot shows the proportion of overall variance explained by each individual principal component (factor).

**Figure 5:**
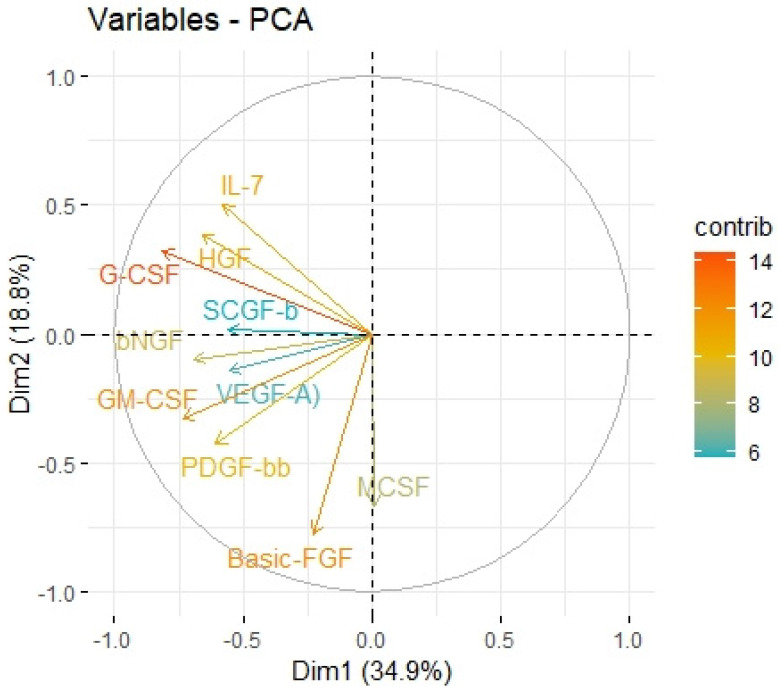
Principal components of protumor growth factors representing variance in two dimensions. PCA loading plot showing PCA of the protumor trophic factors and correlation circle is demonstrated around the PCA. X-axis (Dim1/factor1) is orthogal to y-axis (Dim 2/factor 2). The color and length of vector indicate how strongly each cytokine contributes to principal components/factors (Dim 1 and Dim 2) and how original concentrations of cytokines are associated with their coordinate values.

**Figure 6:**
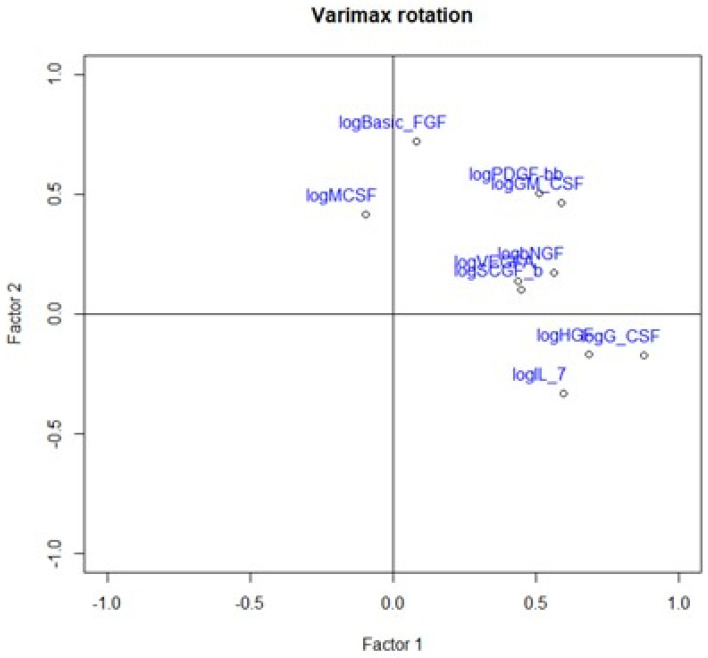
Loading plot presenting loading factors after varimax rotation to change positioning vector of the factors and provide easier interpretation of factor loadings.

**Figure 7:**
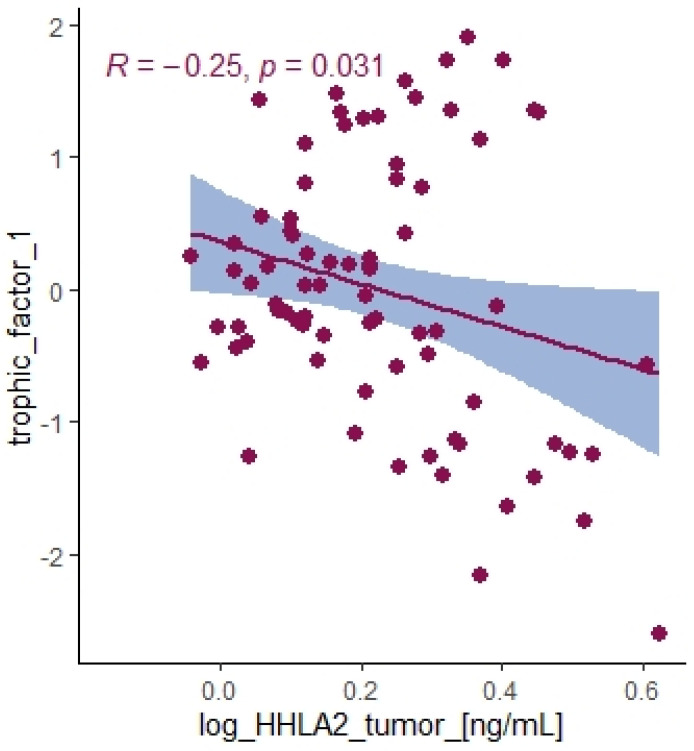
Correlation between HHLA2 tumor concentration and trophic factor1 from principal component analysis containing HGF, M-CSF, b-NGF, SCGF-b, IL-7, FGFbasic, G-CSF, PDGF-bb, VEGF, GM-CSF.

**Figure 8:**
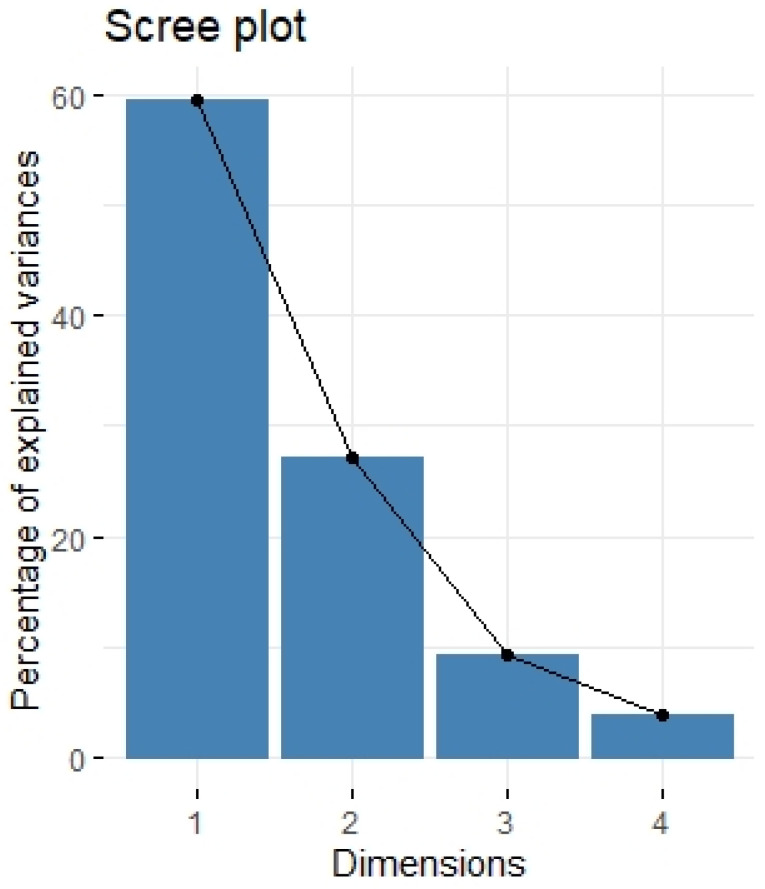
Scree plot of principal component analysis containing antitumor cytokines: IL-1a, IL-12, IL-13, TNF-a. Scree plot shows the proportion of overall variance explained by each individual principal component (factor).

**Figure 9:**
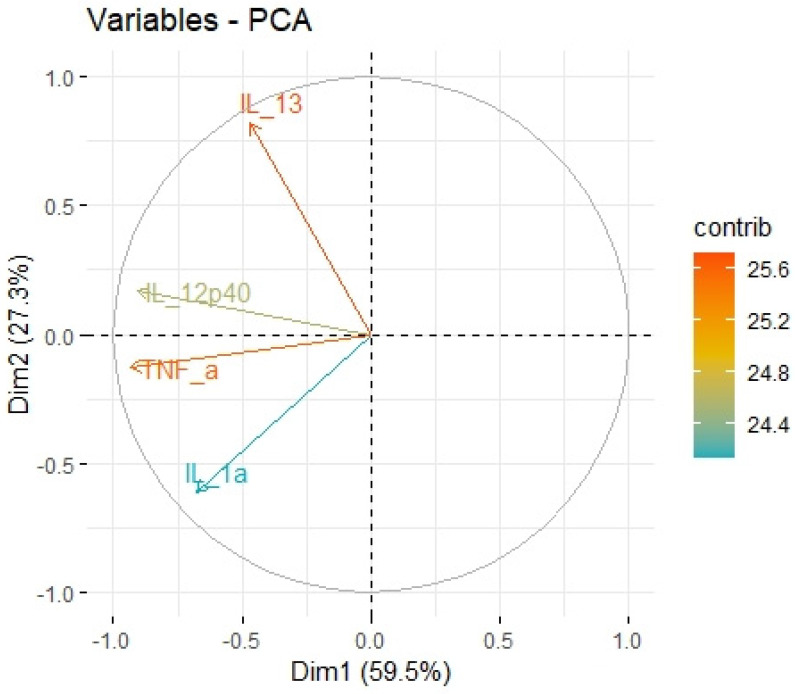
Principal components of antitumor cytokines representing variance in two dimensions. PCA loading plot showing PCA of the antitumor cytokines and correlation circle is demonstrated around the PCA. X-axis (Dim1/factor1) is orthogal to y-axis (Dim 2/factor 2). The color and length of vector indicate how strongly each cytokine contributes to principal components/factors (Dim 1 and Dim 2) and how original concentrations of cytokines are associated with their coordinate values.

**Figure 10:**
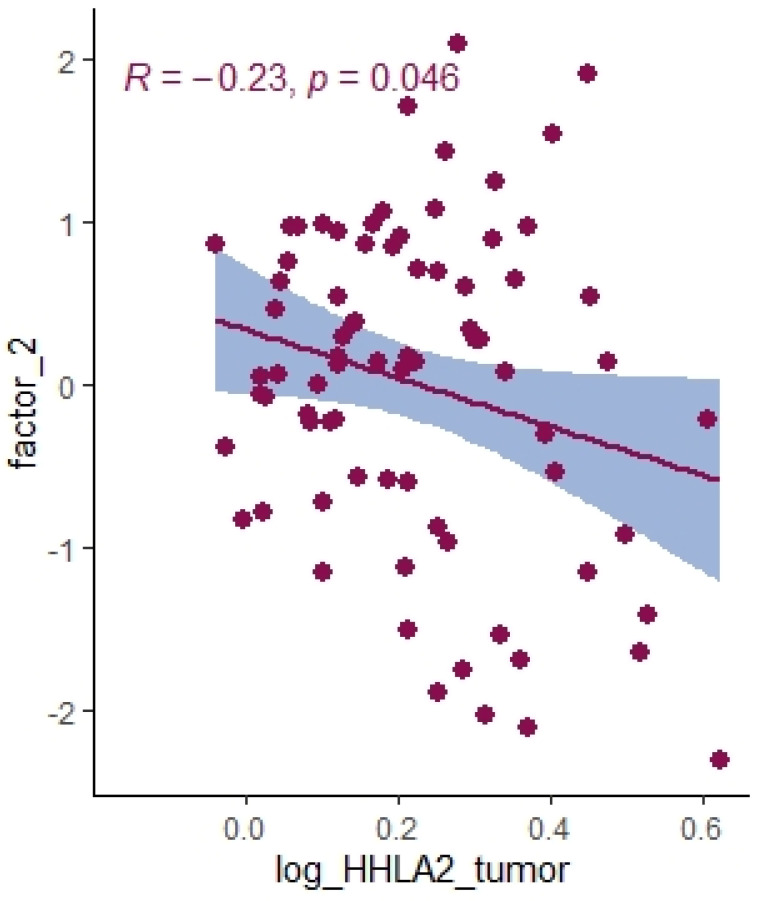
Correlation between HHLA2 tumor concentration factor 2 from principal component analysis containing cytokines IL-1a, IL-12, IL-13, TNF-a.

We divided the Bio-Plex Pro Human Cytokine screening panel into protumor and antitumor immunity groups. First, we performed principal component analysis among protumor growth factors (HGF, M-CSF, b-NGF, SCGF-b, IL-7, FGFbasic, G-CSF, PDGF-bb, VEGF, GM-CSF) and selected cytokines (Il-1a, IL12(p40), IL-13, TNFa). In the section with PCA among protumor growth factors, we presented the Scree plot showing the proportion of overall variance explained by each principal component (HGF, M-CSF, b-NGF, SCGF-b, IL-7, FGFbasic, G-CSF, PDGF-bb, VEGF, GM-CSF) ( Figure 4). Then we presented the PCA loading plot showing PCA of the protumor trophic factors (Figure 5). We changed the positioning vector of the factors via varimax rotation to provide a more straightforward interpretation of factor loadings (Figure 6). PCA parameters characterizing PCA model of protumor cytokines, including loadings of factors after varimax rotation, eigenvalues of factors, and cumulative percentage of overall variance explained by factor 1 and factor 2, are provided in Table 4. Factor 1 complete with factor 2 explain together more than 50% of overall variance. Factor 1 (trophic factor1) obtained from this PCA performed among protumor growth factors (HGF, M-CSF, b-NGF, SCGF-b, IL-7, FGFbasic, G-CSF, PDGF-bb, VEGF, GM-CSF) was negatively correlated with HHLA2 concentrations ( Figure 7). In the second PCA analysis performed among antitumor cytokines (IL-1a, IL-12, IL-13, TNF-a) we also obtained two main components (antitumor factor 1 and antitumor factor 2). The scree plot presenting the proportion of overall variance explained by each principal component is shown in Figure 8. The PCA loading plot showing PCA with correlation circle is provided in Figure 9. PCA parameters characterizing the PCA model of antitumor cytokines, including loadings of factors after varimax rotation, eigenvalues of factors, and cumulative percentage of overall variance explained by antitumor factor 1 and antitumor factor 2, are included in Table 5. In PCA containing antitumor cytokines, factor 1 complete with factor 2 explain together more than 80% of overall variance. Antitumor factor 2 obtained from this PCA correlated negatively with HHLA2 levels (Figure 10).

### 2.4. Immune Infiltration Landscape of HHLA2 Based on the Cancer Genome Atlas-Colon Adenocarcinoma (TCGA-COAD) Data

**Figure 11:**
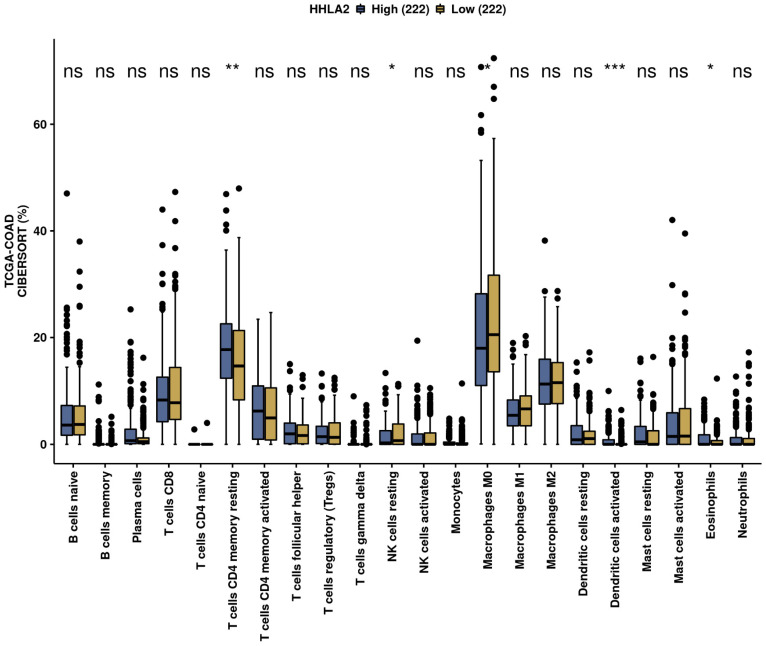
The immunological landscape of CRC related to HHLA2 expression, with analyses based on THE CANCER GENOME ATLAS-COLON ADENOCARCINOMA (TCGA-COAD) dataset. Immune cell infiltration scores calculated by CIBERSORT method by the web-based tool Camoip in high- and low-*HHLA2*-expression groups; * *p* < 0.05, ** *p* < 0.01, and *** *p* < 0.001; ns = not significant.

**Figure 12:**
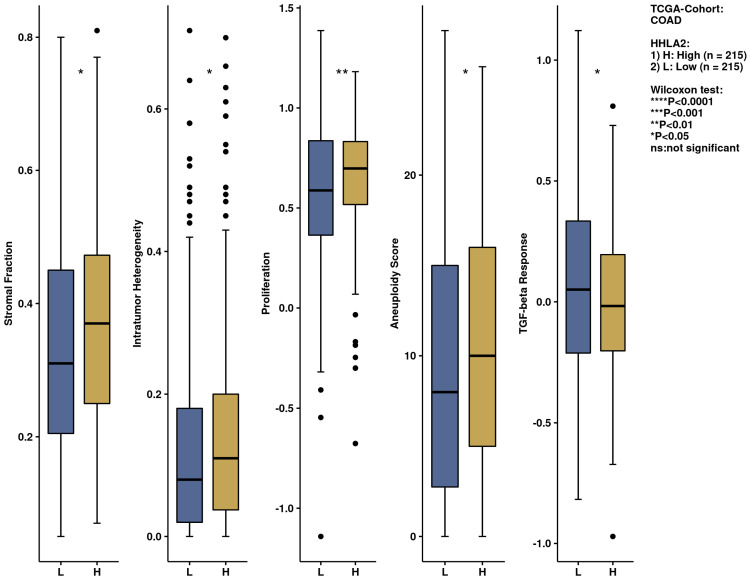
The immunological landscape of CRC related to HHLA2 expression, with analyses based on THE CANCER GENOME ATLAS-COLON ADENOCARCINOMA (TCGA-COAD) dataset. Immune-related scores in high- and low-HHLA2-expression groups, calculated by CIBERSORT method by the web-based tool, Camoip in high and low *HHLA2* expression groups; * *p* < 0.05, ** *p* < 0.01, and *** *p* < 0.001; ns = not significant.

We performed an analysis of the impact of HHLA2 on the immune infiltration landscape in colorectal cancer. Tumor-infiltrating immune cell analysis was calculated by the CIBERSORT algorithm by the web-based tool Comprehensive Analysis on Multi-Omics of Immunotherapy in Pan-cancer (CAMOIP) (Figure 11). The fraction of CD4 resting cells, NK resting cells, macrophages M0, activated dendritic cells, and eosinophils were associated with HHLA2 expression. In addition, the immune scores of stromal fraction, intratumor heterogeneity, proliferation, and aneuploidy score were significantly upregulated in the HHLA2 high-expression group, unlike the TGF-beta response score, which was decreased in this group (Figure 12).

### 2.5. Comprehensive Analysis of Signaling Pathways for HHLA2 Expression

**Figure 13:**
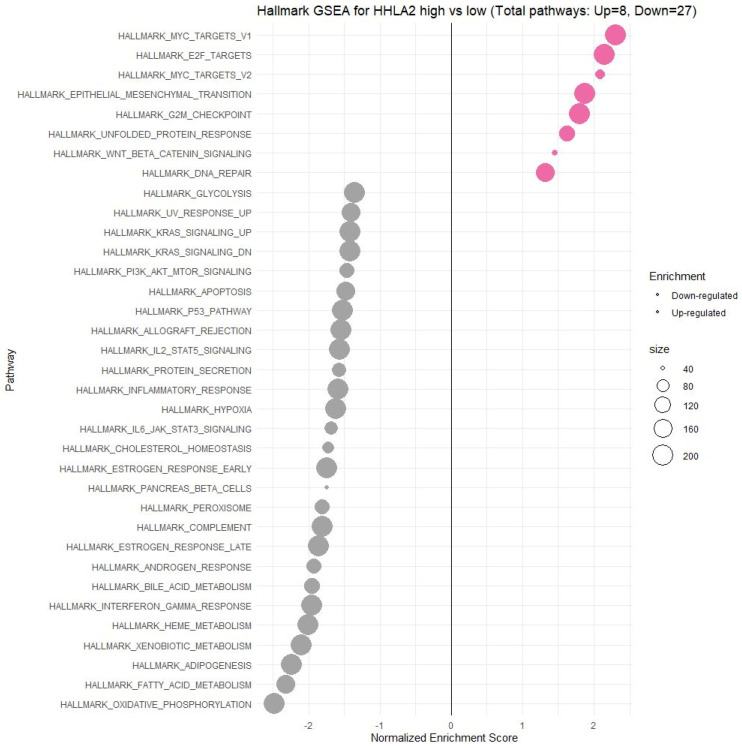
Gene set enrichment analysis (GSEA). The most involved significant hallmark correlated with *HHLA2* in CRC. NES: normalized enrichment score.

**Figure 14:**
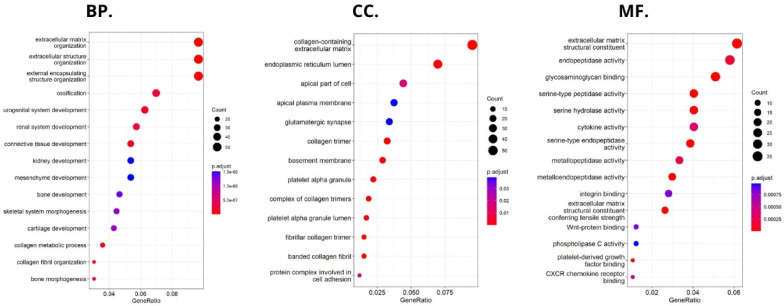
Significantly enriched GO annotations of HHLA2 in FieldEffectCrc dataset: (BP) biological processes; (CC) cellular components; (MF) molecular functions.

HHLA2-related pathways activated in CRC were analyzed by performing GSEA between low and high HHLA2 expression datasets. Significant differences (p.adjusted < 0.05) in the Molecular Signature Database Collection enrichment for hallmark gene sets are shown in Figure 13. The main upregulated pathways for HHLA2 were the c-MYC target pathway, E2F-target pathway, epithelial–mesenchymal transition EMT, and G2M checkpoint. Conversely, the most critical downregulated pathways related to high expression of HHLA2 were the Kras and PI3K/AKT/MTOR signaling pathways and the apoptosis and inflammatory response pathways. The biological function of HHLA2 was predicted by Gene Ontology (GO). The analysis demonstrated that HHLA2 was significantly associated with extracellular matrix structural constituents, endopeptidase activity, and glycosaminoglycan-binding in the molecular function category (MF) and with collagen-containing extracellular matrix in the cellular components category (CC) and extracellular matrix organization, extracellular structure organization, and external encapsulating structure organization in the biological process category (BP) (Figure 14).

## 3. Discussion

Immune-checkpoint blockade therapy (ICBT) has been regarded as an effective cancer treatment method [11]. Several monoclonal antibodies, including nivolumab and pembrolizumab, have been approved to treat CRC [15]. However, there is a large group of patients with clinically advanced CRC whose tumors are resistant to anti-PD-1/PDL1 therapy, especially the patients with microsatellite-stable (MSS) tumors or with low microsatellite instability (MSI-L) [16]. Thus, exploring new targets such as immune checkpoints may increase the efficacy of ICBT.

HHLA2 is a novel immune checkpoint molecule belonging to the B7 family of ligands and is widely expressed in cancer samples and participates in the growth and development of various cancers [13,17]. Our study found a significantly higher level of HHLA2 protein in tumors compared to healthy tissue, and 97% of tumors stained positive for HHLA2. Table 2 presents the division of the examined tumors into HHLA2-positive and HHLA2-negative tumors.

Upregulation of HHLA2 was also demonstrated in other studies considering colorectal cancer. Yang et al. found out that there was an overexpression of HHLA2 mRNA in CRC tissues [13]. Wang et al. concluded that due to its high expression, both at the mRNA and protein levels, HHLA2 may be a potential immunotherapeutic target for CRC patients [18].

Our work is the first study investigating the association between the expression of HHLA2 and MSI/MSS status. We reported no association between HHLA2 expression and MSI/MSS status. However, we observed significant overexpression of HHLA2 both in MSI and MSS tumors (Table 3). Microsatellite-stable (MSS) colorectal cancer has been considered resistant to immunotherapy for a long time due to its lower TMB and less immune infiltration [5]. According to a recent study by Mlecnik et al., some MSS tumors are able to provide an antitumor response; 45% of MSS and 65% of MSI-H colorectal cancers had a high immunoscore, while 55% of MSS and 35% of MSI-H colorectal cancers had a low immunoscore [19] The immunoscore is a predictive marker of response to immunotherapies targeting immune checkpoints [20]. The importance of HHLA2 in tumor genesis and invasion in colorectal cancer remains unclear.

Recently, HHLA2 has been characterized as an immune checkpoint with a dual role in immune responses. The heterogeneity of HHLA2 results from its biological structure and co-inhibitory and costimulatory receptors first reported as transmembrane and immunoglobulin domain–containing 2 (TMIGD2, CD28H, IGPR-1) and recently discovered Killer Cell Immunoglobulin-Like Receptor, Three Ig Domains and Long Cytoplasmic Tail 3 (KIR3DL3). TMIGD2 is a stimulatory receptor of the HHLA2 ligand and is widely expressed on dendritic cells, monocytes, B cells, and naive CD4+ and CD8+ T cells. HHLA2 binding to TMIGD2 promotes T-cell proliferation, differentiation, Nk-cell activation, and cytokine production [21]. In our research, the level of HHLA2 expression correlated positively with TILS (T cells, B cells, natural killer (NK) or dendritic cells (DC) infiltrating the tumor, the primary response of the host immune system) (*p* = 0.019). This result suggests that HHLA2 comprises a costimulatory pathway. These conclusions require confirmation in further research. Furthermore, Yuven et al. identified that TMIGD2 expression gradually decreased with T cell activation and differentiation, and TMIGD2 has been shown to disappear when the naive T cells were activated. This phenomenon may indicate the limited costimulatory role of HHLA2.

The second receptor, Killer Cell Immunoglobulin Like Receptor, Three Ig Domains and Long Cytoplasmic Tail 3 (KIR3DL3), is a member of the KIR family and is an inhibitory receptor of HHLA2 [22]. The KIR3DL3–HHLA2 relation is characterized as a human immunosuppressive pathway and potential therapeutic target to inhibition. Their interaction suppresses T cells and NK cells and reduces cytokine production, including interferon-gamma (IFNγ), TNF-α, IL-5, IL-10, IL-13, IL-17A, and IL-22 [21,23]. Our study did not find a significant relationship between HHLA2 expression level and CD8+ T-cell infiltration. However, GSEA showed a negative relation between HHLA2 and inflammatory response, possibly due to the inhibitory interaction of KIR3DL3–HHLA2. This relation reveals the critical role of HHLA2 in cancer immunity.

Using the FieldEffectCrc dataset, we focused on the functions, mechanisms, and significant common pathways in CRC in relation to HHLA2 expression via GO enrichment analysis and GSEA.

Enrichment analysis revealed that HHLA2 was associated with epithelial–mesenchymal transition (EMT) in CRC. In the process of EMT, cells lose their epithelial characteristics, polarity, and cell–cell contact, gaining mesenchymal properties, such as increased motility, which is a crucial driver of cancer metastasis [24,25,26]. Various experiments showed that HHLA2 overexpression promotes EMT. Expression of EMT markers, such as E-cadherin, N-cadherin, and Vimentin, were significantly changed after the knockdown of HHLA2 expression in human ccRCC [27]. Zhang et al. found out that HHLA2 via an impact on EMT in advanced gallbladder cancer can promote tumor progression [28]. They presented the hypothesis that HHLA2 may be involved in the Wnt/βcatenin axis. The Wnt signaling pathway is one of the critical signaling pathways in regulating cell proliferation and plays a crucial role during the different steps of tumor growth, progression, and metastases [18,21]. According to our GSEA results, there was a relation between HHLA2 and the Wnt/βcatenin pathway, which suggests the impact of HHLA2 in tumor development [21]. 

Moreover, GSEA showed that HHLA2 was linked to critical hallmark pathways in colorectal cancer related to cell cycle regulation and apoptosis. There was a relation between a high expression of HHLA2 and upregulation of “HALLMARK_MYC_TARGETS_V1” and the “HALLMARK_MYC_TARGETS_V2”. MYC is one of the most studied oncogenes known to promote cell proliferation. Several studies have reported a significant correlation between high MYC V1 and V2 scores, worse survival, and cancer aggressiveness [29,30,31,32]. Strippoli et al. demonstrated that higher MYC levels are associated significantly with higher and faster resistance to anti-EGFR chemotherapy treatment. They found a significant association between MYC and lower OS, indicating MYC as a possible negative prognosticator [30]. Chen et al. confirmed in their cellular studies that the MYC expression was significantly decreased after the HHLA2 knockdown [27].

Furthermore, among upregulated pathways linked with HHLA2 high expression were genes critical for the cell cycle and proliferation, such as HALLMARK_E2F_TARGETS, HALLMARK_G2M_CHECKPOINT. Oshi et al. confirmed that the G2M checkpoint pathway was associated with drug response and poor survival in pancreatic cancer [33] and hypothesized that breast cancer tumors with high activity of G2M pathway genes were more aggressive and likely to metastasize [34]. Moreover, E2Fs are crucial regulators of genes required for cell cycle progression and play an integral role in controlling cell proliferation. Importantly, E2Fs control the cell cycle, apoptosis, senescence, DNA damage response, and drug resistance by interacting with multiple signaling pathways [35]. Components regulating the E2F pathway have been identified in nearly every human malignancy and were crucial in cancer progression and metastasis [36]. E2Fs also play a significant role in the regulation of cancer stem cells (CSCs), contributing to various biological characteristics of CSCs, such as proliferation, self-renewal, metastasis, and drug resistance.

Functional analyses of HHLA2 also confirmed its association with tumor progression. Significantly enriched GO terms were tightly associated with invasion, EMT, and tumor microenvironment remodeling. Taken together, the GSEA and GO data suggest that HHLA2 participates in various signaling pathways related to the progression of colorectal cancer. However, any presented relationship requires further research.

We further performed a comprehensive analysis by the web-based tool Camoip and investigated the immune composition of colorectal cancer in relation to HHLA2 expression. We observed an association between various fractions of immune cells, such as CD4+ T cells, NK cells, macrophages, dendritic cells, and HHLA2, which is in line with other studies [21]. The immune scores for stromal fraction, intratumor heterogeneity, proliferation, and aneuploidy were upregulated significantly in the HHLA2 high-expression group (Figure 12), suggesting an impact of HHLA2 on tumorigenesis [37,38].

Using principal component analysis, we received a negative correlation between factor-2-containing cytokines IL-1a, IL-12, IL-13, TNF-a (Table 5) and HHLA2 expression (Figure 10). This result may indicate an interaction between KIR3DL3 and HHLA2 and its inhibitory role in CD4+ and CD8+ T-cell and cytokine production. Research on cytokine production proved that HHLA2, by binding KIR3DL3 on activated T/NK cells, abrogates T-cell proliferation and cytokine production, including IL-5, IL-10, IL-12, IL-13, IL-17A, IL-22, TNF-a, and IFN-g [16,39]. HHLA2 supports tumor development by acting as an inhibitory immune checkpoint through binding with KIR3DL3 in NK and T cells. Obtained results only suggest both the stimulatory and inhibitory impact of HHLA2 on the immune system and require confirmation in further experimental studies.

On the contrary, we found a negative correlation between HHLA2 tumor concentration factor 1 from principal component analysis containing: HGF, M-CSF, b-NGF, SCGF-b, IL-7, FGFbasic, G-CSF, PDGF-bb, VEGF, GM-CSF (Figure 7, Table 4), and trophic factors, which can be produced by colon tumor cells. The investigated factors promote mainly tumor cells and their proliferation and migration. Therefore, the negative correlation between protumor factors and HHLA2 is further evidence of the dual effect of HHLA2 on tumor development.

The critical point of HHLA2 organization is its dual role in an immune response. HHLA2 has two receptors co-inhibitory KIR3DL3, and the costimulatory, TMIGD2. The expression of receptors is determined by the stage of T cell and NK cell activation [21]. Therefore, manipulating the HHLA2-KIR3DL3/TMIGD2 pathway may contribute to the promising strategy of colorectal cancer treatment. Furthermore, KIR3DL3 and TMIGD2 bind simultaneously to different sites of HHLA2, allowing the identification of HHLA2 antibodies that could block the KIR3DL3 inhibitory signal but maintain the TMIGD2 stimulatory signal. Therefore, the specific modulation of one HHLA2 site may be a prospective approach to cancer immunotherapy. Due to the promising idea of the HHLA2/KIR3DL3 blockade without interrupting the HHLA2-TMIDG2 co-immunostimulatory signal, future studies should focus on KIR3DL3–HHLA2 axis blockage without interrupting the costimulatory function [16].

## 4. Materials and Methods

### 4.1. Study Sample

The study involved 167 samples of tumor tissue and surgical margin tissue collected during surgeries due to CRC. The patients were operated on in the 1st Specialist Hospital in Bytom and the Specialist Hospital in Zabrze, Poland (approval of the Research Ethics Committee PCN/0022/KB1/42/VI/14/16/18/19/20). The collected specimens included colorectal tumor tissues and surgical tissue margins. The patients were enrolled in the study after meeting the following criteria: age >18 years, signed written consent, and histological confirmation of colorectal adenocarcinoma and surgical “tumor-free” tissue margin. The exclusion criteria were as follows: no consent to participate in the study, tumors other than adenocarcinoma, tumors with involved margins, and age <18 years. To classify the tumor stage, the TNM staging system and grading were used [40].

The characteristics of the study sample are presented in Table 6.

### 4.2. Evaluation of HHLA2 Level by ELISA

The analysis involved 167 samples of tumor tissue and surgical margin tissue.

The fragments of tumor tissue and surgical margin tissue were weighted and homogenized using a PRO 200 homogenizer (PRO Scientific Inc., Oxford, CT, USA) and sonicated with an ultrasonic cell disrupter (UP 100, Hilscher, Germany). The total protein level was determined using a Universal Microplate Spectrophotometer (µQUANT, Biotek Inc., Winooski, VT, USA).

HHLA2 level was determined by the human HHLA2 ELISA kit (EIAAB SCIENCE INC, WUHAN) with a sensitivity of 0.14 ng/mL. After reaching room temperature by all the reagents, we prepared all the substances, working standards, and samples. The first incubation lasted 2 h at 37 °C after adding 100 uL of standards, blanks, and samples per well. The second incubation lasted 1 h at 37 °C after adding the first detection reagent. After washing processes, we added 100 uL of the second detection reagent, then incubated for 1 h at 37 °C. After washing procedure, 90 uL substrate solution was added and the last incubation lasted 20 min at 37 °C. The last process needed addition of 50 uL stop solution. The optical density of each well was determined using Universal Microplate Spectrophotometer (µQUANT, Biotek Inc., Winooski, VT, USA). (450 nm). The results were recalculated to the corresponding total protein level and presented as ng/mL of protein.

### 4.3. Evaluation of the HHLA2 Expression by IHC

Briefly, 4 µm thick tissue sections were used for immunohistochemical (IHC) analysis. They were deparaffined with xylene, rehydrated in graded alcohol and washed in deionized water. In the next step, antigen retrieval was performed by cooking slides in EnVision Flex Target Retrieval Solution High pH (Dako, Carpinteria, CA, USA) for 20 min at 95 °C. The prepared samples were incubated with Peroxidase-Blocked Reagent (Dako, Carpinteria, CA, USA) and then incubated with the antibody:

HHLA2 Polyclonal Antibody (Dako, Carpinteria, CA, USA) Invitrogen; incubation time, 40′; dilution, 1:300; room temperature.

After this process, they were put in EnVision FLEX HRP (Dako). Then the antigen–antibody complexes were stained using 3,3′-diaminobenzidine. Finally, the tissue sections were counterstained with hematoxylin, dehydrated, and covered with coverslips for further analysis. Histological evaluation was performed by two independent pathologists using an Olympus BX51 microscope. The expression of HHLA2 was evaluated using two parameters: the area of positivity (AP) and the intensity of staining (IS). AP depended on the percentage of positively stained cells. IS was graded as 0 (negative), 1 (weak), 2 (moderate), and 3 (strong). The equation calculated the final H-score: H-score = AP × IS [41].

### 4.4. Assessment of the MSI Status

For MSI/MSS status evaluation the IHC staining for MSH2, MSH6, PMS2, and MLH1 was performed on 4 µm thick sections of a representative formalin-fixed, paraffin-embedded (FFPE) tumor tissue block on a Dako Autostainer Link 48. The samples underwent deparafinization and rehydratation. In the next step, antigen retrieval was performed by cooking slides in EnVision Flex Target Retrieval Solution High pH (Dako, Carpinteria, CA, USA) for 20 min at 95 °C. The prepared samples were incubated with Peroxidase-Blocked Reagent (Dako) and then incubated with one of the following antibodies:

- Mouse Monoclonal Antibody MSH2 (G219-1129), Cell Marque; incubation time, 30′; dilution, 1:400; room temperature.
- Mouse Monoclonal Antibody MSH6 (44), Cell Marque; incubation time, 45′; dilution, 1:100; room temperature.
- Mouse Monoclonal Antibody PMS2 (MRQ-28), Cell Marque; incubation time, 40′; dilution, 1:50; room temperature.
- Mouse Monoclonal Antibody MLH1 (G168-728), Cell Marque; incubation time, 40′; dilution, 1:100; room temperature.

After this process, they were put in EnVision FLEX HRP ((Dako, Carpinteria, CA, USA) Then, the antigen–antibody complexes were stained using 3,3′-diaminobenzidine. Finally, the tissue sections were counterstained with hematoxylin, dehydrated, and covered with coverslips for further analysis.

The tumors were classified as microsatellite-instable by the two pathologists, according to the criteria described by Fassan et al. [42].

### 4.5. Assessment of the Tumor-Infiltrating Cells and Budding

Histological evaluation was performed by two independent pathologists using an Olympus BX51 microscope; 4 µm thick tissue sections were used for immunohistochemical (IHC) analysis. They were deparaffined with xylene, rehydrated in graded alcohol, and washed in deionized water. In the next step, antigen retrieval was performed by cooking slides in EnVision Flex Target Retrieval Solution High pH (Dako, Carpinteria, CA, USA) for 20 min at 95 °C. The prepared samples were incubated with Peroxidase-Blocked Reagent (Dako) and then incubated with antibody:

(CD8/144B) Mouse Monoclonal Antibody diluent; incubation time, 40′; dilution, 1:100; room temperature.

After this process, they were put in EnVision FLEX HRP (Dako). Then the antigen-antibody complexes were stained using 3,3′-diaminobenzidine. Finally, the tissue sections were counterstained with hematoxylin, dehydrated, and covered with coverslips for further analysis.

### 4.6. Assessment of the TILs and Budding

The percentage of tumor-associated lymphatic infiltration was estimated semi-quantitatively on H&E stained slides by the two pathologists, according to the criteria defined by Salgado et al. for breast cancer [43]. These include intratumoral lymphocytes with cell-to-cell contact between the lymphocyte and the tumor cell and stromal TILs in tumor tissue dispersed in the stroma within the tumor cells without direct contact, including TILs at the invasive margin. According to the recommendations, stromal TILs were scored as a percentage of the stromal area alone, excluding areas occupied by carcinoma cells. Lymphatic infiltrates outside the tumor borders were not included in the evaluation. The area of lymphocyte infiltration lower than 5% was considered TILs1 5–25%, 25–50%, and 50–75% of lymphocytes in the stroma were defined as TILs 2, TILs 3, and TILs 4, respectively. More than 75% were defined as TILs 5.

The number of buds was adjusted by the normalization factor (1.210). Budding was reported as follows: low budding, 0–4 buds; intermediate budding, 5–9 buds; high budding, >10 buds. The mean number of buds per FOiV was also used in the statistical analysis.

### 4.7. Bio-Plex Pro Human Cytokines Screening

The concentrations of cytokine/chemokine/growth factors were measured by the Bio-Plex Pro Human cytokine screening panel 48 cytokines assay (Bio-Rad Laboratories, Hercules, CA, USA) according to the manufacturer’s instructions. In brief, a 50 µL aliquot of sample was diluted 1:4 with sample diluent, incubated with antibody-coupled beads, biotinylated secondary antibodies, and followed by streptavidin–phycoerythrin. The beads were read on a Luminex System (Bioplex 200, Bio-Rad), and the data were analyzed using Bioplex Manager Software. The method had been previously used in lymphocyte cell culture supernatants and in blood serum samples [44,45].

### 4.8. Exploration of Biological Characteristics of HHLA2

We conducted annotation analysis based on mRNA expression profiles in CRC online dataset from FieldEffectCrc Package among cohort A, consistent with 311 CRC samples [46]. We normalized the matrix data using DESeq2 package [47]. Then, we divided the study group into high versus low HHLA2 expressions. Gene set enrichment analysis was used to elucidate the potential hallmark pathways from the Molecular Signatures Database (h.all.v7.5.symbols.gmt) of HHLA2 in CRC in R Studio with fgsea package. In this analysis, 3 sections were distinguished: molecular functions (MF), to assess activities that occur at the molecular level and can be performed by HHLA2 gene product; cellular component (CC), to characterize cellular structures in which HHLA2 gene performs a function; and biological processes (BP), to know the processes related to HHLA2 activity. The genes with significant differences in expression among high vs. low HHLA2 expression were screened for GO enrichment analyses (|logFC| > 0.5 and p.adj. < 0.05).

### 4.9. Statistical Analysis

Data distribution was assessed using the Shapiro–Wilk test. The log transformation of the concentrations of the studied proteins provided a better fit for the Gaussian distribution. The data are presented as mean ± SD for the variables with normal distribution and as median with interquartile range for the variables with the non-normal distribution. The paired Student’s *t*-test (for variables with a normal distribution) and Mann–Whitney U test (for non-normal distribution) were used to compare the tumor and margin concentrations. Independent variables were also compared using the Student’s *t*-test and Mann–Whitney U test. Pearson’s coefficient or Spearman coefficient were used to assess the relationships between the examined variables (for variables with normal and non-normal distribution, respectively). Tau: Kendalls’ tau rank correlation coefficient was used to determine the association between the levels of the examined proteins, T, and N parameters. *p* values <0.05 were considered significant. Principal component analysis (PCA) was performed using STATISTICA 13 software (Statsoft) and the package factoextra in R Studio (Integrated Development for R. RStudio, PBC, Boston, MA, USA). PCA aims to change strongly correlated input variables into a coordinate system by using new uncorrelated variables which explain the maximum fraction of variance of input variables. This way, PCA transforms a large set of correlated data into fewer variables called principal components/factors and, and thus reduces the amount of data. In our analyses, we used a two-component PCA model in which input variables (cytokines concentrations) are presented in a two-dimensional vector space with the largest fraction of explained variance distributed in two DIMS/factors, with DIM1/Factor 1 on the x-axis, and Dim2/Factor 2 on *y*-axis. The distribution of each cytokine in the PCA model depends on the correlation between the initial concentrations of cytokines and factors obtained in PCA. Positively associated variables create a familiar group, while negatively correlated ones are grouped in quadrants of a plot that is diagonally opposed. Component axes were rotated using a varimax method to fit positioning vectors better and simplify the factor loading interpretation. The factors obtained in principal component analysis were used as new variables, named trophic factor 1 and trophic factor 2 (for PCA performed among protumor growth factors), and various cytokines, 1 and 2 (for PCA performed among Il-1a, IL12(p40), IL-13, and TNFa, according to their proportions of explained variance). Next, the obtained factors’ values were used in further analyses. *p* values ≤0.05 were considered significant. All other statistical analysis was performed using STATISTICA 13 software (Statsoft) and the R Studio (Integrated Development for R. RStudio, PBC, Boston, MA, USA).

## 5. Conclusions

In conclusion, we demonstrated that HHLA2 is upregulated in patients with CRC, and we were the first ones to report significant overexpression of HHLA2 in MSI and MSS tumors. HHLA2 was involved significantly in critical hallmark pathways related to EMT, cell cycle regulation, apoptosis, and immune regulation.

We conducted principal component analysis demonstrating evidence of the dualistic effect of HHLA2 on tumor development. High expression of HHLA2 correlated positively with TILS, suggesting a limited costimulatory role of HHLA2. We revealed the role of HHLA2 expression as a stimulatory and inhibitory immune checkpoint in colorectal cancer, highlighting that the HHLA2–KIR3DL3/TMIGD2 pathway may contribute to the promising strategy of colorectal cancer treatment. Further studies must elucidate the mechanisms of HHLA2 overexpression and its therapeutic values in colorectal cancer.

## Figures and Tables

**Table 1:** HHLA2 concentrations in tumor and margin [pg/mL], N = 167. Paired T-test.

HHLA2 Concentration	Mean	SD	*p*
Log HHLA2 tumor	0.17	0.16	<0.0001
Log HHLA2 margin	0.05	0.14	

**Table 2:** HHLA2 Immunohistochemistry expression in tumor cells (N = 77).

Characteristics	HHLA2 Tumor Expression	
all	positive	negative
77 (100%)	75 (97.40%)	2 (2.60%)

**Table 3:** Association between HHLA2 IHC expression and MSI/MSS status of tumors. Chi-square test.

MSS/MSI Status	HHLA2 Expression		
	Negative	Positive	*p*
MSS	1 (1.49%)	66 (98.51%)	0.2
MSI	1 (10%)	9 (90%)	

**Table 4:** Loadings of factors after varimax rotation.

Variable	Factor1	Factor2
logHGF	0.686	−0.169
logMCSF		0.415
logbNGF	0.565	0.172
logSCGF-b	0.451	
logIL-7	0.597	−0.332
logBasic-FGF		0.722
logG-CSF	0.880	−0.173
logPDGF-bb	0.513	0.506
logVEGFA	0.437	0.139
logGM-CSF	0.590	0.463
eigenvalue	3.488	1.876
variance.percent	34.883	18.761
cumulative.variance.percent	34.883	53.645

**Table 5:** Loadings of factors after varimax rotation.

Variable	Factor1	Factor2
log(IL-1a_tumor/protein)	0.890	−0.211
log(IL-12p40_tumor/protein)	0.718	0.580
logIL-13_tumor/protein)	0.018	0.944
log(TNF-a-tumor/protein)	0.880	0.335
eigenvalue	2.084	1.386
variance.percent	52.11	34.67
cumulative.variance.percent	52.11	87.78

**Table 6:** Patients characteristics.

	Female	Male	All Cases
	76 (45.51%)	91 (54.49%)	167 (100%)
Age	66.63 ± 9.48	63.75 ± 9.46	65.09 ± 9.55
Tumor localization			
Left-sided	49 (66.22%)	65 (73.86%)	114 (70.37%)
Right-sided	25 (33.78%)	23 (26.13%)	48 (29.63%)
T parameter			
T1	1 (1.33%)	7 (8.05%)	8 (4.94%)
T2	16 (21.33%)	12 (13.79%)	28 (22.22%)
T3	47 (62.67%)	54 (62.07%)	101 (62.35%)
T4	11 (14.67%)	14 (16.09%)	25 (15.43%)
N parameter			
N0	32 (42.67%)	37 (42.05%)	69 (42.33%)
N1	30 (40.00%)	37 (42.05%)	67 (41.10%)
N2	13 (17.33%)	14 (15.91%)	27 (16.56%)
M parameter			
M0	66 (88.00%)	69 (79.31%)	135 (83.33%)
M1	9 (12.00%)	18 (20.69%)	27 (16.67%)
TNM stage			
I	13 (17.33%)	13 (14.77%)	26 (15.95%)
II	19 (25.33%)	21 (28.86%)	40 (24.54%)
III	34 (45.33%)	37 (42.05%)	71 (43.55%)
IV	9 (12.00%)	17 (19.32%)	26 (15.95%)
Grading			
Low	63 (85.14%)	75 (85.23%)	138 (85.16%)
High	11 (14.86%)	13 (14.777%)	24 (14.81%)
HHLA2 IHC expression			
Positive	34 (97.14%)	41 (97.62%)	77 (97.40%)
Negative	1 (2.86%)	1 (2.38%)	2 (2.60%)
MSS/MSI status (N = 101)			
MSS tumors	36 (76.60%)	45 (83.33%)	81 (80.20%)
MSI tumors	11 (23.40%)	9 (16.67%)	20 (19.80%)
TILs (N = 102)			
0–5%	16 (34.04%)	30 (53.5%)	46 (44.66%)
6–25%	15 (31.91%)	13 (23.21%)	28 (27.18%)
26–50%	14 (29.79%)	8 (14.29%)	22 (21.36%)
51–75%	2 (4.26%)	4 (7.14%)	6 (5.83%)
76–100%	0 (0.00%)	1 (1.79%)	1 (0.97%)
CD8+ Lymphocytes (N = 76)			
0–5%	14 (40.00%)	20 (47.62%)	34 (44.16%)
6–25%	8 (22.86%)	13 (30.95%)	21 (71.43%)
26–50%	7 (20.00%)	5 (11.90%)	12 (87.01%)
51–75%	4 (11.43%)	2 (4.76%)	6 (7.79%)
76–100%	2 (5.71%)	2 (4.76%)	4 (5.19%)
Budding (N = 101)			
0–4	28 (59.57%)	29 (53.70%)	57 (56.44%)
5–9	13 (27.66%)	11 (20.37%)	24 (23.76%)
>9	6 (12.77%)	14 (25.93%)	20 (19.80%)
Adjuvant treatment			
yes	7 (9.21%)	14 (15.38%)	21 (12.35%)
no	69 (90.79%)	77 (84.62%)	149 (87.65%)

## Data Availability

Not Applicable.
